# Myxozoan survey of thicklip grey mullet *Chelon labrosus* reinforces successful radiation of *Myxobolus* in mugiliform hosts[Fn FN1]

**DOI:** 10.1051/parasite/2023029

**Published:** 2023-07-04

**Authors:** José Guimarães, Graça Casal, Ângela Alves, Catarina Araújo, Sónia Rocha

**Affiliations:** 1 ICBAS – School of Medicine and Biomedical Sciences, University of Porto, Rua Jorge Viterbo Ferreira no. 228 4050-313 Porto Portugal; 2 TOXRUN – Toxicology Research Unit, University Institute of Health Sciences, CESPU, CRL 4585-116 Gandra Portugal; 3 Faculty of Sciences (FCUP), University of Porto Rua do Campo Alegre, s/n, FC4 4169-007 Porto Portugal; 4 Instituto de Investigação e Inovação em Saúde (i3S), University of Porto Rua Alfredo Allen no. 208 4200-135 Porto Portugal

**Keywords:** Diversity, Morphological plasticity, Geographic isolates, 18S rDNA, Life cycle, Sphaeractinomyxon

## Abstract

A myxozoan survey was performed on specimens of thicklip grey mullet *Chelon labrosus* (Risso) captured from the Douro River estuary, northern Portugal. Eleven new species, all belonging to the genus *Myxobolus* Bütschli, 1882 (*M. abdominalis* n. sp., *M. aestuarium* n. sp., *M. caudalis* n. sp., *M. chelonari* n. sp., *M. cucurbitiformis* n. sp., *M. douroensis* n. sp., *M. intestinicola* n. sp., *M. invictus* n. sp., *M. labicola* n. sp., *M. peritonaei* n. sp., and *M. pinnula* n. sp.) are described based on microscopic and molecular data, confirming the known high radiation of these myxozoans in mullets. Additionally, *Myxobolus pupkoi* Gupta *et al*., 2022 is reported for the first time from *C. labrosus*, bringing forth a novel case of morphological plasticity between geographic isolates. We consider that molecular-based comparisons are imperative for the description of mugiliform-infecting *Myxobolus*, with distance estimation further matching two of the novel *Myxobolus* spp. with sphaeractinomyxon types previously reported from another Portuguese estuary. This finding supports sphaeractinomyxon as specific life cycle counterparts of *Myxobolus* that infect mullets. Phylogenetic analyses of 18S rDNA retrieved a monophyletic clade of mugiliform-infecting myxobolids comprising well-supported lineages of species parasitizing mullets from the genera *Chelon*, *Mugil*, *Crenimugil*, and *Planiliza*. The existence of more than one *Chelon*- and *Planiliza*-infecting lineage reveals that myxobolids parasitized members of these genera multiple times during their evolution. Lastly, the elevated number of unmatched sphaeractinomyxon sequences included in the *Chelon*-infecting lineages clearly shows that *Myxobolus* diversity hosted by this genus remains underrated.

## Abstract (fr)

Un inventaire des myxozoaires a été réalisé sur des spécimens de mulets lippus *Chelon labrosus* (Risso) capturés dans l’estuaire du fleuve Douro, au nord du Portugal. Onze nouvelles espèces, toutes appartenant au genre *Myxobolus* Bütschli, 1882 (*M. abdominalis* n. sp., *M. aestuarium* n. sp., *M. caudalis* n. sp., *M. chelonari* n. sp., *M. cucurbitiformis* n. sp., *M. douroensis* n. sp., *M. intestinicola* n. sp., *M. invictus* n. sp., *M. labicola* n. sp., *M. peritonaei* n. sp. et *M. pinnula* n. sp.) sont décrites sur la base de données microscopiques et moléculaires, confirmant le rayonnement connu de ces myxozoaires chez les mulets. De plus, *Myxobolus pupkoi* Gupta *et al*., 2022 est signalé pour la première fois chez *C. labrosus*, démontrant un nouveau cas de plasticité morphologique entre des isolats géographiques. Nous considérons que les comparaisons moléculaires sont impératives pour la description des *Myxobolus* infectant les mugiliformes, l’estimation de la distance correspondant en outre à deux des nouveaux *Myxobolus* spp. avec des types de sphaeractinomyxons précédemment signalés dans un autre estuaire portugais. Cette découverte soutient les sphaeractinomyxons en tant que contreparties spécifiques du cycle de vie de *Myxobolus* qui infectent les mulets. Les analyses phylogénétiques de l’ADNr 18S ont montré un clade monophylétique de Myxobolidae infectant les mugiliformes, comprenant des lignées robustes d’espèces parasitant les mulets des genres *Chelon*, *Mugil*, *Crenimugil* et *Planiliza*. L’existence de plusieurs lignées infectant *Chelon* et *Planiliza* révèle que les Myxobolidae ont parasité des membres de ces genres plusieurs fois au cours de leur évolution. Enfin, le nombre élevé de séquences de sphaeractinomyxons non appariées incluses dans les lignées infectant *Chelon* montre clairement que la diversité de *Myxobolus* hébergée par ce genre reste sous-estimée.

## Introduction

Mugilids (Teleostei, Mugilidae) are highly opportunistic and diverse fishes that inhabit a wide range of habitats worldwide; because they have a high tolerance to varying abiotic conditions like salinity, temperature, sedimentary regimes, turbidity, and dissolved oxygen [46], mullets can be found in coastal areas, estuaries, lakes, and lagoons, in both tropical and temperate regions. This ubiquitous nature and extreme adaptability grant them excellent survival skills and allows them to perform important roles in ecosystems; they are key players in the flow of matter and energy from lower to higher trophic levels [1, 5, 25]. It also leaves them vulnerable and exposed to parasitic infections and disease.

Myxozoa Grassé, 1970 is a subphylum of cnidarians that evolved to become obligate endoparasites of invertebrates and vertebrates [36]. Myxozoan parasites are also ubiquitous/widespread in aquatic environments, existing both in fresh and saltwater habitats [30, 36]. Myxozoan species are traditionally described using morphological characters as the main taxonomic criteria. However, descriptions based solely on morphology have been proved to be unreliable due to the extreme diversity of this group and high plasticity of myxospores between and within genera [2, 3, 17, 18]. In fact, the molecular analyses of several gene markers have revealed discrepancies between morphology and molecular-based classifications, showing that morphological characters are not enough and may even be misleading when it comes to genus and species-level classification [2, 19, 28, 39, 40]. Therefore, a more integrative approach is required for performing reliable species identifications, which should be based not only on morphology, but also DNA sequencing, host identification, habitat, tissue tropism, histopathology, and other details of the sporogonic stages and their development [2, 19].

Mullets are highly susceptible to infection by myxozoan parasites, with over 90 species identified from these hosts worldwide, most of which belonging to the genus *Myxobolus* Bütschli, 1882 [8, 21, 37, 39, 40, 45, 52]. In fact, Rocha *et al*. [40] reported hyperdiversification of *Myxobolus* spp. in mullet hosts, which presently account for more than 55 species of this myxozoan genus [8, 21, 37, 39, 40, 45, 52]. In Portugal, a total of 14 *Myxobolus* spp. have been reported from mullets, all from the Minho River estuary in the north: 8 from thinlip grey mullet *Chelon ramada* (Risso, 1827); 2 from thicklip grey mullet *Chelon labrosus* (Risso, 1827); and 4 from flathead grey mullet *Mugil cephalus* Linnaeus, 1758 [39, 40]. Another myxozoan, *Ellipsomyxa mugilis* (Sitjà-Bobadilla and Alvarez-Pellitero, 1993), was further reported from *C. ramada*, also in the Minho River estuary [40].

The present study aimed to acknowledge the myxozoan diversity infecting *C. labrosus* in another northern Portuguese estuary (Douro River), contributing to the general knowledge of myxozoan morphological and molecular data, as well as life cycle clarification.

## Materials and methods

### Fish sampling and myxozoan survey

Thirteen specimens of thicklip grey mullet *C. labrosus* (Risso, 1927) were acquired fresh from commercial stocks captured by local fishermen in the Douro River estuary, near the city of Porto (41°08′N8°39′W), during March 2022. Examination of the fish’s external (fins, eyes, skin, and scales) and internal organs and tissues (brain, gills, heart, gonads, liver, stomach, intestines, spleen, gallbladder, swim bladder, urinary bladder, kidney, and muscle) was performed both macroscopically and microscopically, using an Olympus BX41 microscope (Olympus, Coimbra, Portugal) for the detection of myxospores, plasmodia, cysts, or other developmental stages of Myxozoa. Cysts and infected tissues were examined and photographed using an Olympus DP72 digital camera (Olympus). Measurements were taken from fresh mature myxospores, following the guidelines of Lom and Arthur [29]. All measurements are given in micrometers (μm), and include mean ± standard deviation, range of variation (minimum and maximum), and number of myxospores measured (*n*). The prevalence (%) of infection was determined based on matching of the 18S rDNA sequences obtained for each case isolate in study.

### DNA extraction, amplification, and sequencing

After being photographed, cysts and infected tissue samples were separately preserved in absolute ethanol at 4 °C. DNA was extracted with the extraction kit “GenElute^TM^ Mammalian Genomic DNA Miniprep” (Sigma-Aldrich, St Louis, MO, USA). PCRs targeted the amplification of the small subunit ribosomal gene (18S rDNA), using both universal and myxozoan specific primers (Table 1). PCR reactions and cycling conditions were set-up according to Rocha *et al*. [40]. Amplification was confirmed by electrophoresis in a 1% agarose 1× tris-acetate-EDTA buffer (TAE) gel stained with GreenSafe Premium (NZYTech, Lisbon, Portugal). PCR products were purified using the ExoFast method and sequenced directly using a BigDye Terminator v1.1 from the Applied Biosystems kit (Applied Biosystems, Carlsbad, CA, USA), and ABI 3700 DNA analyzer (Perkin-Elmer, Waltham, MA, USA; Applied Biosystems, Carlsbad, CA, USA; Stabvida, Oeiras, Portugal).

**Table 1:** Polymerase chain reaction primers used for amplification and sequencing of the 18S rDNA.

Name	Sequence (5′–3′)	Paired with	Source
18E	CTG GTT GAT CCT GCC AGT	ACT3r, ACT2r	[23]
ACT2f	CCT GGT CCG AAC ATC CGA AGG ATA C	18R	[40]
ACT3f	CAT GGA ACG AAC AAT	18R	[22]
ACT5f	TGT GCC TTG AAT AAA T	18R	[43]
ACT6f	TGA GGA TAG GCA TTG ACC TT	18R	This study
ACT8f	GCC TTG AGT AAA TCA G	18R	This study
ACT2r	GTA TCC TTC GGA TGT TCG GAC CAG G	18E	[40]
ACT3r	ATT GTT CGT TCC ATG	18E	[41]
18R	CTA CGG AAA CCT TGT TAC G	ACT2f, ACT3f, ACT5f, ACT6f, ACT8f	[50]

### Sequence assembly, distance estimation, and phylogenetic analysis

The obtained forward and reverse sequence segments belonging to each sample were separately aligned using ClustalW in MEGA11 software [35, 48]. For distance estimation, a dataset was generated that comprised all newly obtained sequences, plus all sequences available for other mugiliform-infecting *Myxobolus*, sphaeractinomyxon types, and the triactinomyxon type of Székely *et al*., 2007 (DQ473515). This dataset included all sequences with highest similarity score to the newly obtained sequences (above the 80.0% threshold), as determined through BLASTn (Basic Local Alignment Search Tool). Alignments were performed using MAFFT version 7 online, and distance estimation was carried out in MEGA 11, with the *p*-distance model and all ambiguous positions removed for each sequence pair.

The dataset used for phylogenetic analyses also included all newly obtained sequences, plus 53 representatives of mugiliform-infecting *Myxobolus* and sphaeractinomyxon types, and the sequences of *Myxobolus khaliji* (KC711053), *Myxobolus miyairii* (KT001495) and *Henneguya cynoscioni* (JN017203) included as outgroup. Alignments of this dataset were also performed using MAFFT version 7 online. Bayesian inference analyses were conducted in MrBayes v.3.2.6 [44], with the general time reversible model with gamma-shaped rate variations across sites (Invgamma) (GTR + I + G). Posterior probabilities were calculated using the Markov chain Monte Carlo method, in which four chains ran simultaneously for 1 million generations, with burn-in set at 25%, and trees sampled every 500 generations. Maximum likelihood was performed in MEGA11, for 1,000 replicates, with the general time reversible substitution model with estimates of invariant sites and gamma distributed among site rate variation (GTR + I + G) chosen as the best suited model, based on the lowest score of the Bayesian Information Criterion (BIC) and corrected Akaike Information Criterion (AIC).

## Results

### Myxozoan survey and overall prevalence of infection

The myxozoan survey conducted in this study revealed the presence of myxospores either dispersed in the tissue samples or contained within cysts. Of the 13 specimens of *C. labrosus* examined, 10 were infected (76.9%) with *Myxobolus* (Myxosporea, Myxobolidae); infections by other myxozoan genera were not observed. Most specimens were found to be parasitized by more than one *Myxobolus* species, with myxospores present in different organs (see Table 2). Highest species diversity and overall prevalence of infection occurred in the gills, followed by the peritoneum and fins. Co-infection was determined in the swim bladder of specimen#2 and in the fins of specimen#13, based on the analysis of ABI chromatograms, which included numerous overlapping peaks, and suspect base-calling errors in regions other than at the extremities. The species infecting the kidney of specimen#7 failed to be sequenced due to the low amount of parasite DNA (Table 2).

**Table 2:** *Myxobolus* infection in the organs of the 13 specimens of *C. labrosus* examined, as determined by light microscopic observations. Mpin *M. pinnula* n. sp.; Mcau *M. caudalis* n. sp.; Mpup *Myxobolus pupkoi* Gupta *et al.*, 2022; Mche *M. chelonari* n. sp.; Maes *M. aestuarium* n. sp.; Minv *M. invictus* n. sp.; Mdou *M. douroensis* n. sp.; Mlab *M. labicola* n. sp.; Mper *M. peritonaei* n. sp.; Mabd *M. abdominalis* n. sp.; Mcuc *M. cucurbitiformis* n. sp.; Mint *M. intestinicola* n. sp.; M unidentified *Myxobolus* spp. PI: overall prevalence of infection per organ.

Specimen#	Fins	Mouth	Gills	Peritoneum	Gallbladder	Swim bladder	Kidney	Stomach/intestine
1	–	–	–	–	–	–	–	Mint
2	–	–	Mpup	–	–	M	–	–
3	Mpin	–	Mche	–	–	–	–	–
4	–	–	Mpup	–	–	–	–	–
5	–	–	–	Mper	–	–	–	–
7	–	–	–	–	–	–	M	–
9	–	–	Maes	Mper	–	–	–	–
10	Mcau	–	Minv	Mper	–	–	–	–
11	–	–	Mdou	–	Mcuc	–	–	–
12	Mpin	Mlab	Maes	Mabd	–	–	–	–
13	M	–	–	Mabd	–	–	–	–
PI	30.8%	7.7%	53.8%	38.5%	7.7%	7.7%	7.7%	7.7%

**Figure 1:**
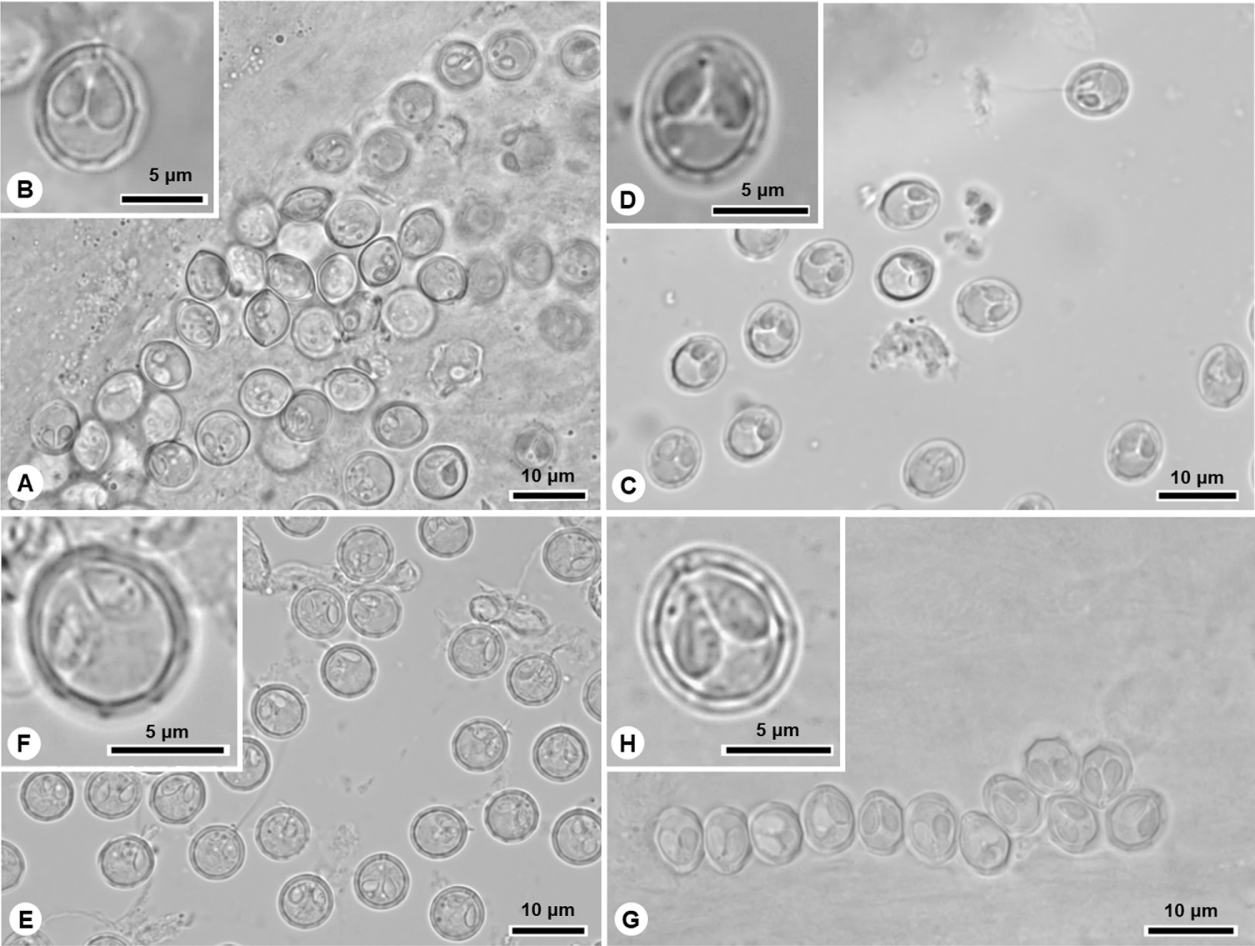
Light micrographs of *Myxobolus* spp. infecting the thicklip grey mullet *Chelon labrosus* in the Douro River estuary. (A)–(B) *M. pinnula* n. sp., (C)–(D) *M. caudalis* n. sp., (E)–(F) *M. pupkoi*, (G)–(H) *M. chelonari* n. sp.

**Figure 2:**
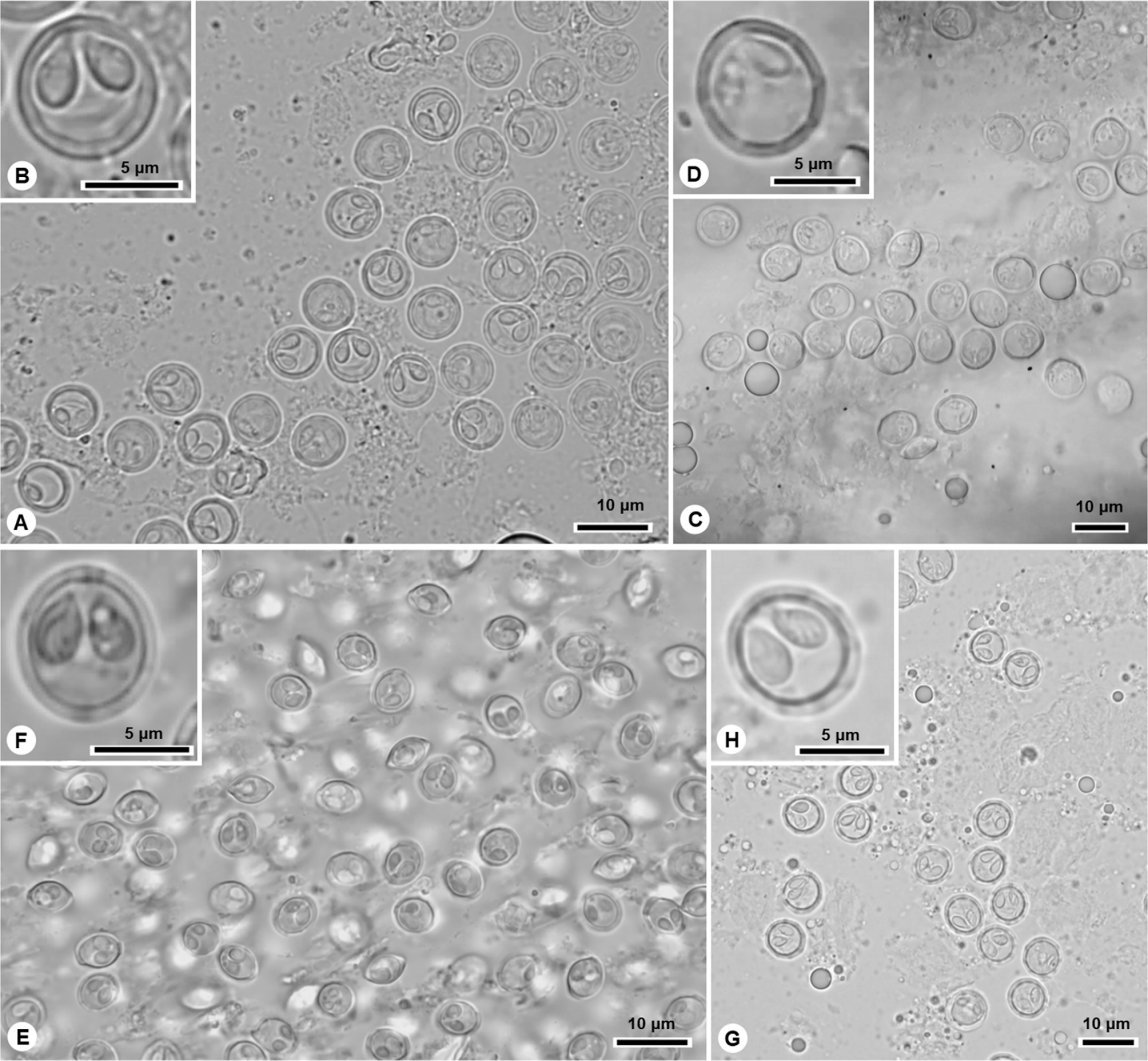
Light micrographs of *Myxobolus* spp. infecting the thicklip grey mullet *Chelon labrosus* in the Douro River estuary. (A)–(B) *M. aestuarium* n. sp., (C)–(D) *M. invictus* n. sp., (E)–(F) *M. douroensis* n. sp., (G)–(H) *M. labicola* n. sp.

**Figure 3:**
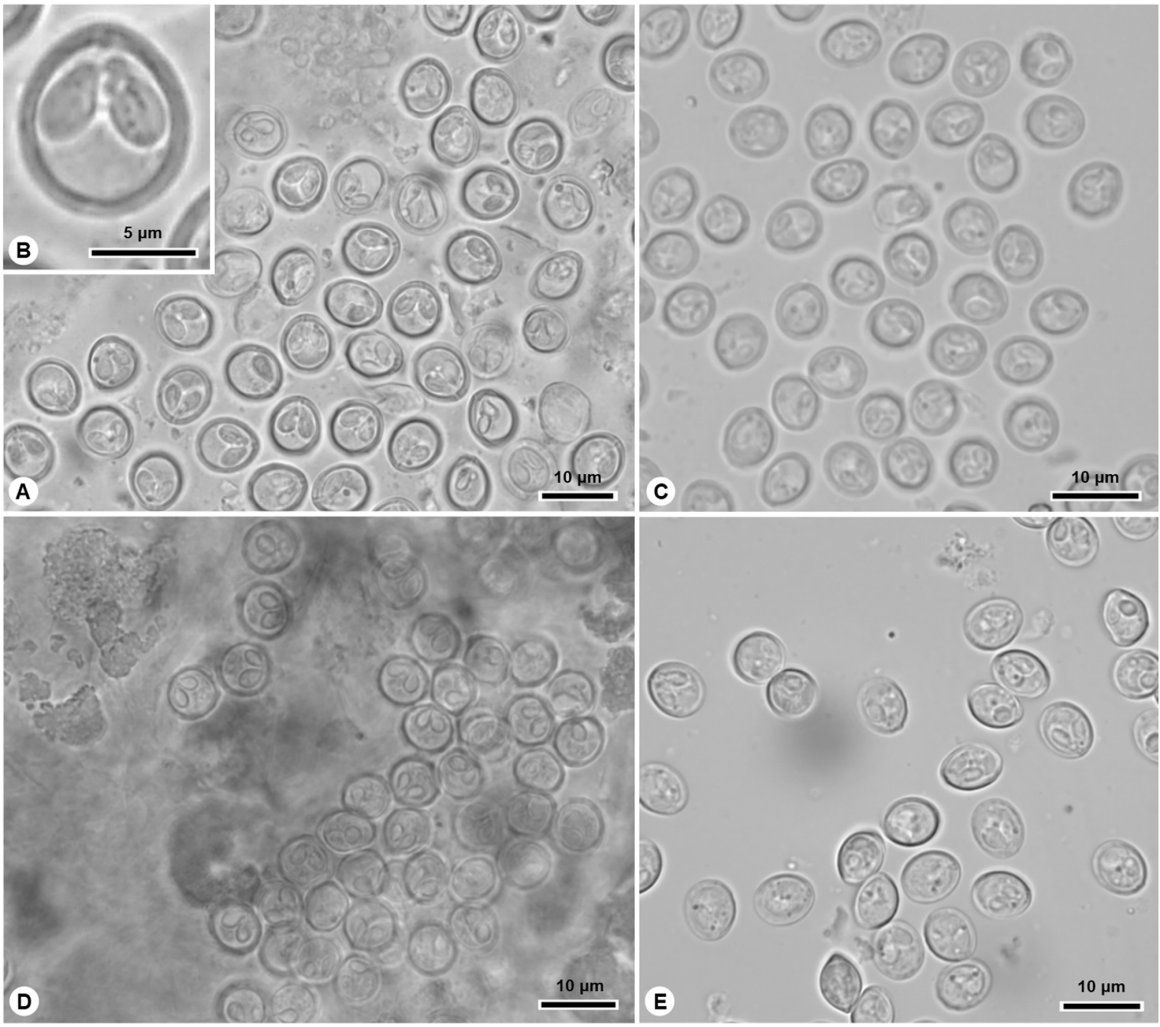
Light micrographs of *Myxobolus* spp. infecting thicklip grey mullet *Chelon labrosus* in the Douro River estuary. (A)–(B) *M. peritonaei* n. sp., (C) *M. abdominalis* n. sp., (D) *M. cucurbitiformis* n. sp., (E) *M. intestinicola* n. sp.

**Figure 4:**
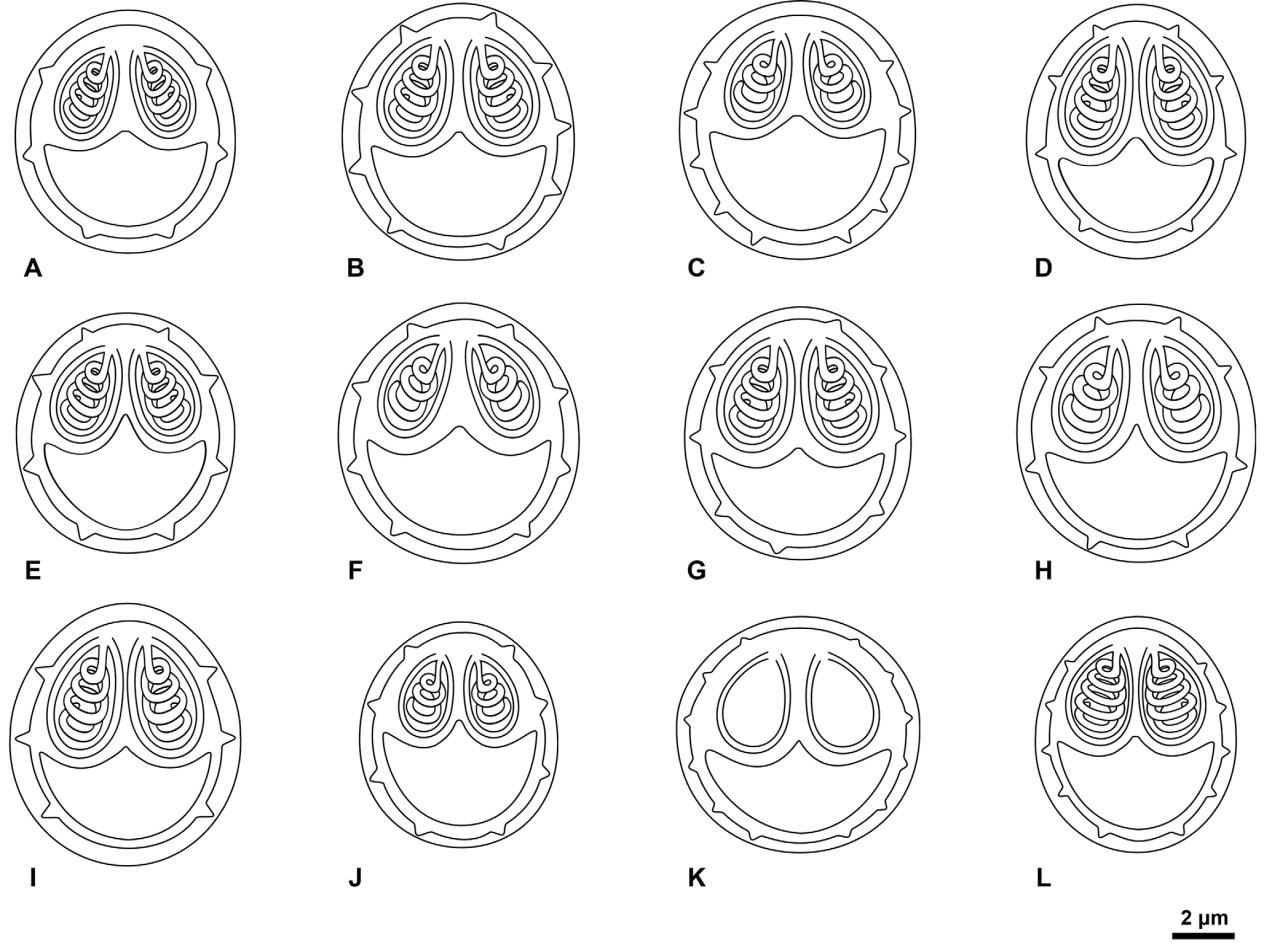
Schematic drawings of the 11 new *Myxobolus* spp. found infecting the thicklip grey mullet *Chelon labrosus* in the Douro River estuary, as observed in valvular view. (A) *M. pinnula* n. sp., (B) *M. caudalis* n. sp., (C) *M. pupkoi*, (D). *M. chelonari* n. sp., (E) *M. aestuarium* n. sp., (F) *Myxobolus invictus* n. sp., (G) *Myxobolus douroensis* n. sp., (H) *M. labicola* n. sp., (I) *M. peritonaei* n. sp., (J) *M. abdominalis* n. sp., (K) *M. cucurbitiformis* n. sp., (L) *M. intestinicola* n. sp.

In this study, eleven new species of *Myxobolus* are described (Figs. 1–4; Table 3), and a novel host, geographic region and morphometric profile is reported for *Myxobolus pupkoi* Gupta *et al*., 2022.

**Table 3:** Myxospore morphometry of the *Myxobolus* spp. described here from *Chelon labrosus* and morphologically similar species.

*Myxobolus* spp.	SL	SW	ST	PCL	PCW	PTt	Source
*M. pinnula* n. sp.	8.1 ± 0.3 (7.3–8.8) (*n* = 60)	7.5 ± 0.4 (6.8–8.6) (*n* = 60)	5.2 ± 0.3 (4.7–5.9) (*n* = 51)	3.3 ± 0.4 (2.5–4.5) (*n* = 60)	2.0 ± 0.2 (1.7–2.6) (*n* = 60)	4	This study
*M. caudalis* n. sp.	8.4 ± 0.2 (8.0–8.9) (*n* = 60)	7.5 ± 0.5 (6.8–8.4) (*n* = 60)	5.3 ± 0.3 (5.1–5.9) (*n* = 7)	3.6 ± 0.3 (3.1–4.2) (*n* = 60)	2.3 ± 0.4 (1.9–4.1) (*n* = 60)	3–5	This study
*M. chelonari* n. sp.	8.3 ± 0.2 (7.7–8.7) (*n* = 30)	7.1 ± 0.4 (6.0–7.5) (*n* = 30)	–	3.9 ± 0.2 (3.5–4.5) (*n* = 30)	2.3 ± 0.1 (1.9–2.4) (*n* = 30)	3–4	This study
*M. aestuarium* n. sp	7.8 ± 0.3 (7.3–8.3) (*n* = 81)	7.1 ± 0.7 (6.0–8.0) (*n* = 63)	5.1 ± 0.3 (4.4–5.8) (*n* = 59)	3.4 ± 0.2 (3.0–3.9) (*n* = 70)	2.1 ± 0.2 (1.8–2.4) (*n* = 69)	3-4	This study
*M. invictus* n. sp	8.5 ± 0.2 (8.1–9.0) (*n* = 30)	7.9 ± 0.2 (7.4–8.2) (*n* = 30)	–	3.6 ± 0.2 (2.3–4.1) (*n* = 22)	2.0 ± 0.1 (1.9–2.3) (*n* = 21)	3	This study
*M. douroensis* n. sp	8.2 ± 0.3 (7.5–9.2) (*n* = 90)	7.3 ± 0.2 (6.5–7.9) (*n* = 90)	5.6 ± 0.2 (5.0–6.2) (*n* = 81)	3.9 ± 0.2 (3.3–4.5) (*n* = 90)	2.5 ± 0.2 (2.0–3.0) (*n* = 90)	3–4	This study
*M. labicola* n. sp	8.4 ± 0.2 (8.1–8.7) (*n* = 30)	7.8 ± 0.2 (7.4–8.3) (*n* = 30)	5.9 ± 0.2 (5.4–6.2) (*n* = 30)	4.0 ± 0.2 (3.5–4.4) (*n* = 30)	2.4 ± 0.1 (2.1–2.6) (*n* = 30)	3	This study
*M. peritonaei* n. sp	8.8 ± 0.4 (8.0–9.6) (*n* = 120)	7.7 ± 0.3 (6.9–8.4) (*n* = 120)	5.9 ± 0.5 (5.0–7.1) (*n* = 79)	3.8 ± 0.4 (3.0–4.6) (*n* = 105)	2.2 ± 0.2 (1.8–2.8) (*n* = 105)	4	This study
*M. abdominalis* n. sp	7.1 ± 0.3 (6.5–7.7) (*n* = 60)	6.1 ± 0.4 (5.2–6.8) (*n* = 60)	4.8 ± 0.3 (4.2–5.5) (*n* = 49)	2.7 ± 0.2 (2.1–3.3) (*n* = 60)	1.6 ± 0.2 (1.3–2.0) (*n* = 60)	3	This study
*M. cucurbitiformis* n. sp	7.7 ± 0.3 (7.0–8.4) (*n* = 30)	7.9 ± 0.4 (7.1–8.8) (*n* = 30)	–	3.4 ± 0.3 (2.8–4.2) (*n* = 30)	2.3 ± 0.2 (1.8–2.8) (*n* = 30)	–	This study
*M. intestinicola* n. sp.	7.7 ± 0.3 (7.1–8.1) (*n* = 30)	6.5 ± 0.3 (5.8–7.2) (*n* = 30)	5.6 ± 0.2 (5.2–5.8) (*n* = 6)	3.4 ± 0.3 (2.8–3.9) (*n* = 30)	2.1 ± 0.2 (1.8–2.5) (*n* = 30)	4–5	This study
*M. pupkoi* Gupta *et al.*, 2022	8.4 ± 0.3 (7.9–9.7) (*n* = 60)	7.7 ± 0.4 (7.0–8.7) (*n* = 60)	5.1 ± 0.3 (4.5–5.5) (*n* = 20)	3.2 ± 0.2 (2.8–4.0) (*n* = 60)	2.0 ± 0.2 (1.6–2.4) (*n* = 60)	3–4	This study
	5.8 ± 0.1 (5.7–6.2)	5.4 ± 0.1 (5.3–5.5)	–	2.4 ± 0.2 (2.3–2.7)	1.3 ± 0.2 (1.1–1.8)	4–5	[21]
*M. adeli* (Isjumova, 1964) Yurakhno and Ovcharenko, 2014	6.2 ± 0.3 (5.6–6.8)	7.2 ± 0.3 (6.6–7.8)	4.6 ± 0.4 (3.5–5.3)	3.1 ± 0.3 (2.4–3.8)	1.8 ± 0.2 (1.3–2.3)	4	[52]
*M. cheni* Schulman, 1962	8.0 – 8.5	6.0–6.5	–	4.5–5.0	2.0	–	[12]
*M. parsi* Das, 1996	9.1 (9.0–9.5)	8.1 (8.0–8.5)	–	4.4 (4.0–4.5)	2.8 (2.5–3.0)	5	[9]
*M. hani* Faye *et al.*, 1999	8.0 ± 0.5 (7.0–9.1)	7.3 ± 0.3 (7.0–8.0)	–	–	–	–	[15]
*M. macropeli* Dorothy and Kalavati, 1992	6.2 (5.2–6.9)	5.3 (4.3–6.9)	–	2.8 (1.7–3.4)	2.0 (1.7–2.5)	6-7	[10]
*Myxobolus* sp*.*1	6.9 ± 0.3 (6.3–7.3)	5.9 ± 0.5 (4.7–6.7)	4.2 ± 0.2 (4.0–4.7)	3.1 ± 0.2 (2.7–3.3)	1.8 ± 0.2 (1.3–2.0)	–	[40]
*Myxobolus* sp*.*2	7.1 ± 0.4 (6.5–8.0)	5.5 ± 0.3 (5.1–6.0)	4.3 ± 0.3 (3.8–4.7)	3.1 ± 0.3 (2.6–3.6)	2.1 ± 0.2 (1.6–2.4)	3–4	[40]
*Myxobolus* sp.3	8.9 ± 0.4 (7.9–9.6)	7.9 ± 0.4 (7.1–8.5)	5.5 ± 0.3 (5.2–6.1)	4.5 ± 0.2 (4.0–5.0)	2.7 ± 0.2 (2.5–3.2)	6	[40]
*Myxobolus* sp.5	7.8 ± 0.4 (7.2–8.8)	7.5 ± 0.4 (6.5–8.5)	5.5 ± 0.3 (5.1–6.0)	L 4.7 ± 0.4 (3.9–5.4)	L 3.3 ± 0.2 (3.0–3.8)	L 8	[40]
				S3.6±0.2(3.3–4.2)	S2.1±0.2(1.6–2.4)	S 6	

SL: myxospore length; SW: myxospore width; ST: myxospore thickness; PCL: polar capsule length; PCW: polar capsule width; PTt: number of turns of the polar tubule; L: larger; S: smaller.

### *Myxobolus pinnula* n. sp.

urn:lsid:zoobank.org:act:C32A9530-0C6C-4C1B-855A-F7D436636EB2

Diagnosis: Round whitish cysts measuring < 1 mm, located in fins. Myxospores spherical in valvular view and ellipsoidal in sutural view, with ca. six sutural edge markings positioned equidistant at posterior pole. Two pyriform and equally sized polar capsules located side by side at anterior pole, each containing polar tubule coiled in four turns (Figs. 1A, 1B, 4A, Table 3).

Site of infection: Fins.

Type locality: Douro River estuary (41°08′N8°39′W), near the city of Porto, Portugal.

Prevalence of infection: 2 infected of 13 specimens examined (15.4%) (Table 2).

Material deposited: Series of phototypes of the hapantotype, deposited together with a representative DNA sample in the Natural History and Science Museum of the University of Porto, Portugal, reference CIIMAR.2023.72.

Etymology: The specific epithet “*pinnula*” is a noun referring to the site of infection.

Molecular data: One 18S rDNA sequence with 1,985 nucleotides and GenBank accession number OQ319166, representative of two identical sequences that were separately obtained from both infected specimens. Distance estimation revealed highest similarity to the sequences of *M. pupkoi* (OL605966, OQ319154) (98.4%) and *M. caudalis* n. sp. (97.9%), with all other sequences included in the analysis displaying similarity values lower than 97.3%.

### *Myxobolus caudalis* n. sp.

urn:lsid:zoobank.org:act:D1B69DB6-6CE0-48B7-869B-5E5AFA9A4A6C

Diagnosis: White spherical cysts, 1–2 mm in size, located between rays of caudal fin. Myxospores spherical to subspherical in valvular view and ellipsoidal in sutural view, with 6–10 sutural edge markings. Two large equally sized pyriform polar capsules located side by side at anterior pole, occupying a little less than half myxospore body, each containing polar tubule coiled in 3–5 turns (Figs. 1C, 1D, 4B, Table 3).

Site of infection: Caudal fin.

Type locality: Douro River estuary (41°08’N8°39’W), near the city of Porto, Portugal.

Prevalence of infection: 1 infected of 13 specimens examined (7.7%) (Table 2).

Material deposited: Series of phototypes of the hapantotype, deposited together with a representative DNA sample in the Natural History and Science Museum of the University of Porto, Portugal, reference CIIMAR.2023.73.

Etymology: The specific epithet “*caudalis*” refers to the site of infection.

Molecular data: One 18S rDNA sequence with 1,934 nucleotides and GenBank accession number OQ319156. Distance estimation revealed highest similarity to the sequences of *M. pupkoi* (OL605966, OQ319154) (98.0-98.1%) and *M. pinnula* n. sp. (97.9%), with all other sequences included in the analysis displaying less than 96.7% similarity to the isolate.

### *Myxobolus pupkoi* Gupta *et al*., 2022

Diagnosis: Macroscopic whitish cysts measuring about 1 mm, located in gills. Myxospores spherical to subspherical in valvular view, and ellipsoidal in sutural view, with ca. 10 sutural edge markings surrounding valves. Two pyriform polar capsules, equally sized, located side by side at anterior pole, each containing polar tubule coiled in 3–4 turns (Figs. 1E, 1F, 4C, Table 3).

Site of infection: Gills.

Localities: Sea of Galilee (31°49′N35°38′E) (Gupta *et al*., 2022) (type locality); Douro River estuary (41°08′N8°39′W), near the city of Porto, Portugal.

Prevalence of infection: 2 infected of 13 specimens examined (15.4%) (Table 2).

Material deposited: Series of phototypes of the hapantotype, deposited together with a representative DNA sample in the Natural History and Science Museum of the University of Porto, Portugal, reference CIIMAR.2023.74.

Molecular data: One 18S rDNA sequence with 2,006 nucleotides and GenBank accession number OQ319154, representative of two identical sequences that were separately obtained from both infected specimens. Distance estimation revealed highest similarity to the sequence of *M. pupkoi* (OL605966) (99.3%), *M. pinnula* n. sp. (98.4%) and *M. caudalis* n. sp. (98.0%), with all other sequences included in the analysis displaying similarity values lower than 97.1%.

### *Myxobolus chelonari* n. sp.

urn:lsid:zoobank.org:act:1F53415C-66D4-4C91-908E-4E9422602E0F

Diagnosis: Macroscopic cysts measuring about 1 mm, located in gills. Myxospores ovoid in valvular view, with 8–9 sutural edge markings surrounding valves. Two equally sized pyriform polar capsules, occupying more than half myxospore body, located side by side at anterior pole, each with polar tubule coiled in 3–4 coils (Figs. 1G, 1H, 4D, Table 3).

Site of infection: Gills.

Type locality: Douro River estuary (41°08’N8°39’W), near the city of Porto, Portugal.

Prevalence of infection: 1 infected of 13 specimens examined (7.7%) (Table 2).

Material deposited: Series of phototypes of the hapantotype, deposited together with a representative DNA sample in the Natural History and Science Museum of the University of Porto, Portugal, reference CIIMAR.2023.75.

Etymology: The specific epithet “*chelonari*” refers to the host genus.

Molecular data: One 18S rDNA sequence with 1,968 nucleotides and GenBank accession number OQ319218, representative of five identical sequences that were separately obtained from 5 cysts collected from a single infected specimen. Distance estimation revealed highest similarity to *M. peritonaei* n. sp. (98.1%) and *M. invictus* n. sp. (97.8%), with all other sequences included in the analysis displaying similarity values lower than 96.9%.

### *Myxobolus aestuarium* n. sp.

urn:lsid:zoobank.org:act:7D5C63AE-2AB7-4EAB-B5CB-625A22E37558

Diagnosis: Macroscopic cysts measuring less than 2 mm, located in gills. Myxospores spherical to subspherical in valvular view and ellipsoidal in sutural view, with 6–10 sutural edge markings surrounding valves. Two pyriform and equally sized polar capsules located side by side at anterior pole, each displaying polar tubule coiled in four turns (Figs. 2A, 2B, 4E, Table 3).

Site of infection: Gills.

Type locality: Douro River estuary (41°08′N8°39′W), near the city of Porto, Portugal.

Prevalence of infection: 2 infected of 13 specimens examined (15.4%) (Table 2).

Material deposited: Series of phototypes of the hapantotype, deposited together with a representative DNA sample in the Natural History and Science Museum of the University of Porto, Portugal, reference CIIMAR.2023.76.

Etymology: The specific epithet “*aestuarium*” is a noun referring to the estuarine habitat of the host species.

Molecular data: One 18S rDNA sequence with 1,972 nucleotides and GenBank accession number OQ319169, representative of two identical sequences that were separately obtained from both infected specimens. Distance estimation did not reveal similarity values higher than 96.6% to all other sequences reported here or retrieved from the NCBI database.

### *Myxobolus invictus* n. sp.

urn:lsid:zoobank.org:act:53024C10-99AA-4FE8-A0EC-3D96DB72BEA8

Diagnosis: Macroscopic cysts ~1 mm, located in gills. Myxospores spherical to subspherical in valvular view, with 6–8 sutural edge markings surrounding valves. Two pyriform and equally sized polar capsules located side by side at anterior pole, each displaying polar tubule coiled in three turns (Figs. 2C, 2D, 4F, Table 3).

Site of infection: Gills.

Type locality: Douro River estuary (41°08′N8°39′W), near the city of Porto, Portugal.

Prevalence of infection: 1 infected of 13 specimens examined (7.7%) (Table 2).

Material deposited: Series of phototypes of the hapantotype, deposited together with a representative DNA sample in the Natural History and Science Museum of the University of Porto, Portugal, reference CIIMAR.2023.77.

Etymology: The specific epithet “*invictus*” refers to the city of Porto, historically known as “*Invicta*” (meaning: the invincible city).

Molecular data: One 18S rDNA sequence with 1,953 nucleotides and GenBank accession number OQ319224. Distance estimation did not reveal similarity values higher than 97.9% to all other sequences reported here or retrieved from the NCBI database.

### *Myxobolus douroensis* n. sp.

urn:lsid:zoobank.org:act:B0CA7FF0-0E69-480B-88B9-273B783B5AF1

Diagnosis: Round whitish macroscopic cysts (~1 mm) adjacent to gill filaments. Myxospores subspherical in valvular view and ellipsoidal in sutural view, with 6–12 sutural edge markings surrounding valves. Two pyriform and equally sized polar capsules located side by side at anterior pole, each displaying polar tubule coiled in 3–4 turns (Figs. 2E, 2F, 4G, Table 3).

Site of infection: Gills.

Type locality: Douro River estuary (41°08′N8°39′W), near the city of Porto, Portugal.

Prevalence of infection: 1 infected of 13 specimens examined (7.7%) (Table 2).

Material deposited: Series of phototypes of the hapantotype, deposited together with a representative DNA sample in the Natural History and Science Museum of the University of Porto, Portugal, reference CIIMAR.2023.78.

Etymology: The specific epithet “*douroensis*” refers to the type locality, which is the Douro River.

Molecular data: One 18S rDNA sequence with 1,989 nucleotides and GenBank accession number OQ319165. Distance estimation did not reveal similarity values higher than 90.5% to all other sequences reported here or retrieved from the NCBI database.

### *Myxobolus labicola* n. sp.

urn:lsid:zoobank.org:act:DA6D235A-759F-4ECB-8CB7-FFCB902E22B4

Diagnosis: Several clustered whitish macroscopic cysts, forming clover-shaped cyst with ~3/4 mm inside protrusible top lip (mouth). Myxospores spherical in valvular view and subspherical in sutural view, with 8–10 sutural edge markings surrounding valves. Two pyriform and equally sized polar capsules located side by side at anterior pole, each displaying polar tubule coiled in three turns (Figs. 2G, 2H, 4H, Table 3).

Site of infection: Protrusible top lip (mouth).

Type locality: Douro River estuary (41°08′N8°39′W), near the city of Porto, Portugal.

Prevalence of infection: 1 infected of 13 specimens examined (7.7%) (Table 2).

Material deposited: Series of phototypes of the hapantotype, deposited together with a representative DNA sample in the Natural History and Science Museum of the University of Porto, Portugal, reference CIIMAR.2023.79.

Etymology: The specific epithet “*labicola*” refers to the site of infection.

Molecular data: One 18S rDNA sequence with 1,968 nucleotides and GenBank accession number OQ319221. Distance estimation did not reveal similarity values higher than 93.0% to all other sequences reported here or retrieved from the NCBI database.

### *Myxobolus peritonaei* n. sp.

urn:lsid:zoobank.org:act:949C484F-A35E-43FB-AE01-2CC5D4CCDCE5

Diagnosis: Subspherical to elongate whitish cysts measuring 1–2 mm in length, located in peritoneum. Myxospores spherical to subspherical in valvular view, with 5–6 sutural edge markings located at posterior pole. Two equally sized pyriform polar capsules located side by side at anterior pole, each containing polar tubule coiled in ca. four turns (Figs. 3A, 3B, 4I, Table 3).

Site of infection: Peritoneum.

Type locality: Douro River estuary (41°08′N8°39′W), near the city of Porto, Portugal.

Prevalence of infection: 3 infected of 13 specimens examined (20.0%) (Table 2).

Material deposited: Series of phototypes of the hapantotype, deposited together with a representative DNA sample in the Natural History and Science Museum of the University of Porto, Portugal, reference CIIMAR.2023.80.

Etymology: The specific epithet “*peritonaei*” refers to the site of infection.

Molecular data: One 18S rDNA with 2,002 nucleotides and GenBank accession number OQ319164, representative of five identical sequences that were separately obtained from cysts collected from three infected specimens. Distance estimation revealed highest similarity to *M. chelonari* n. sp. (98.1%) and *M. invictus* n. sp. (97.9%), with all others displaying less than 97.0% similarity.

### *Myxobolus abdominalis* n. sp.

urn:lsid:zoobank.org:act:7994843E-485F-4AB4-ACD0-F5D05E8C3B98

Diagnosis: Round whitish macroscopic cysts (~1 mm) adjacent to mesentery, spread throughout abdominal cavity. Myxospores subspherical in valvular view and lemon-shaped in sutural view, with 8 sutural edge markings surrounding valves. Two small pyriform and equally sized polar capsules located side by side at anterior pole, each displaying polar tubule coiled in three turns (Figs. 3C, 4J, Table 3).

Site of infection: Peritoneum.

Type locality: Douro River estuary (41°08′N8°39′W), near the city of Porto, Portugal.

Prevalence of infection: 2 infected of 13 specimens examined (15.4%) (Table 2).

Material deposited: Series of phototypes of the hapantotype, deposited together with a representative DNA sample in the Natural History and Science Museum of the University of Porto, Portugal, reference CIIMAR.2023.81.

Etymology: The specific epithet “*abdominalis*” refers to the distribution of the parasite in the host abdominal cavity.

Molecular data: One 18S rDNA sequence with 1,982 nucleotides and GenBank accession number OQ319155, representative of two identical sequences that were separately obtained from both infected specimens. Distance estimation did not reveal similarity values higher than 93.3% to all other sequences reported here or retrieved from the NCBI database.

### *Myxobolus cucurbitiformis* n. sp.

urn:lsid:zoobank.org:act:D77202F1-62CD-4A7E-B599-19E517CD3E7F

Diagnosis: Groups of myxospores distributed throughout gallbladder tissue. Myxospores pumpkin-shaped in valvular view, with greater width than length, and ellipsoidal in sutural view, with 10–12 sutural edge markings surrounding valves. Two pyriform equally sized polar capsules located side by side at anterior pole (Figs. 3D, 4K, Table 3).

Site of infection: Gallbladder.

Type locality: Douro River estuary (41°08′N8°39′W), near the city of Porto, Portugal.

Prevalence of infection: 1 infected of 13 specimens examined (7.7%) (Table 2).

Material deposited: Series of phototypes of the hapantotype, deposited together with a representative DNA sample in the Natural History and Science Museum of the University of Porto, Portugal, reference CIIMAR.2023.82.

Etymology: The specific epithet “*cucurbitiformis*” refers to the pumpkin-shape of the myxospores.

Molecular data: One 18S rDNA sequence with 1,988 nucleotides and GenBank accession number OQ319220. Distance estimation matched the newly obtained sequence with that of Sphaeractinomyxon type 5 of Rangel *et al*. [29] (KU569314) (99.8%), with all other sequences included in the analysis displaying similarity values lower than 91.1%.

### *Myxobolus intestinicola* n. sp.

urn:lsid:zoobank.org:act:649C3445-B741-492E-A3D2-553C534134D9

Diagnosis: Macroscopic cysts located in intestine. Myxospores subspherical, almost ellipsoidal in valvular view, ellipsoidal in sutural view, with more than nine sutural edge markings surrounding valves. Two pyriform and equally sized polar capsules located side by side at anterior pole, each displaying polar tubule coiled in 4–5 turns (Figs. 3E, 4L, Table 3).

Site of infection: Intestine.

Type locality: Douro River estuary (41°08′N8°39′W), near the city of Porto, Portugal.

Prevalence of infection: 1 infected of 13 specimens examined (7.7%) (Table 2).

Material deposited: Series of phototypes of the hapantotype, deposited together with a representative DNA sample in the Natural History and Science Museum of the University of Porto, Portugal, reference CIIMAR.2023.83.

Etymology: The specific epithet “*intestinicola*” refers to the site of infection.

Molecular data: One 18S rDNA sequence with 1,963 nucleotides and GenBank accession number OQ319222. Distance estimation matched the newly obtained sequence with that of Sphaeractinomyxon type 8 of Rangel *et al*. [29] (KU569317) (99.9%), with all other sequences included in the analysis displaying similarity values lower than 95.6%.

### Differential diagnosis

Differentiation between the novel isolates reported in this study relied on the comparison of their 18S rDNA sequences, given that few significant morphometrically distinguishable features could be found between myxospores (see Table 3). Morphological comparisons were performed in relation to all *Myxobolus* spp. that were previously reported from mullet hosts and that are without molecular data.

All isolates reported in this study shared some morphometric similarity with *Myxobolus hani* Faye *et al*., 1999 (see Table 3), which could be readily differentiated based on different organ of infection (branchial spines) whenever compared to species infecting other organs, host genus (*Mugil curema* Valenciennes, 1836), geographic location (Atlantic Ocean off Senegal), and slight differences in terms of length and width [15]. Myxospores of *M. hani* have a slightly smaller width range than *M. pinnula* n. sp. and *M. caudalis* n. sp., and slightly greater length range than *M. caudalis* n. sp., *M. invictus* n. sp., *M. douroensi*s n. sp. and *M. labicola* n. sp. In comparison to *M. chelonari* n. sp. and *M. aestuarium* n. sp., the myxospores of *M. hani* are thinner with a slightly greater length range, while being shorter than those of *M. peritonaei* n. sp., wider than those of *M. abdominalis* n. sp. and *M. intestinicola* n. sp., and having a greater width range than those of *M. cucurbitiformis* n. sp.

Except for *M. aestuarium* n. sp., *M. abdominalis* n. sp. and *M. intestinicola* n. sp., all other isolates further showed some morphometric similarity to *Myxobolus cheni* Schulman, 1962 (see Table 3), which could be promptly distinguished based on organ of infection (trunk muscle), host species [*M. cephalus*, and *Planiliza haematocheilus* (Temminck and Schlegel, 1845)], geographic location (China), and overall smaller myxospore width and longer polar capsules, also with slight differences in the length range for most cases [12].

*Myxobolus peritonaei* n. sp. further resembled *Myxobolus parsi* Das, 1996 (see Table 3) that, however, differs by having myxospores with a smaller length and width range, different organ of infection (gills), host species [*Chelon parsia* (Hamilton, 1822)], and geographic location (India) [9]. In turn, *M. abdominalis* n. sp. shared some morphometric similarity with *Myxobolus adeli* (Isjumova, 1964) Yurakhno and Ovcharenko, 2014 reported from several organs of *Chelon auratus* (Risso, 1810) in the Mediterranean Sea off Spain, Azov and Black Seas, and *Myxobolus macrolepi* Dorothy and Kalavati, 1992 from the intestine of *Planiliza macrolepis* (Smith, 1846) in India (see Table 3). Apart from these differences in organ of infection, host species and geographic location, the myxospores of *M. adeli* are larger with a greater thickness range and display three polar tubule coils instead of four, while those of *M. macrolepsi* are shorter with a greater width range, further displaying a higher number of polar tubule coils (6–7) [10, 52].

Comparison with unnamed *Myxobolus* further revealed that most species described here share some morphometric similarity with the *Myxobolus* sp. 3 and *Myxobolus* sp. 5 reported by Rocha *et al*. [40] from *C. ramada*, with the first differing by having overall larger polar capsules with a higher number of polar tubule coils (6), and the latter differing by having unequally sized polar capsules. The myxospores of *M. abdominalis* n. sp. further resembled the *Myxobolus* sp. 1 and *Myxobolus* sp. 2 of Rocha *et al*. [40] with only slight morphometric variations, while those of *M. intestinicola* n. sp., which also shared some similar features with *Myxobolus* sp. 2 of Rocha *et al*. [40], could be differentiated based on the latter being less thick (see Table 3).

Overall, the comprehensive comparison of morphological, molecular, host-related, and geographic data performed in this study supported the description of these isolates as new species. The only exception was *M. pupkoi*, originally described from the gill arches of *C. ramada* in the Galilee Sea off Israel [21], and that was here identified from the gills of *C. labrosus* based on a 99.3% similarity score. This small genetic difference (0.7%) mostly took place at the 3′ end of the 18S rDNA sequence, with a few nucleotide differences occurring elsewhere (position 694: T *vs.* A; positions 1508–1509: C *vs.* Y; and position 1538: A *vs.* W). Morphometric comparisons revealed significant differences among our isolates of *M. pupkoi* and the isolate reported by Gupta *et al*. [21] (see Table 3).

### Phylogenetic analysis

**Figure 5:**
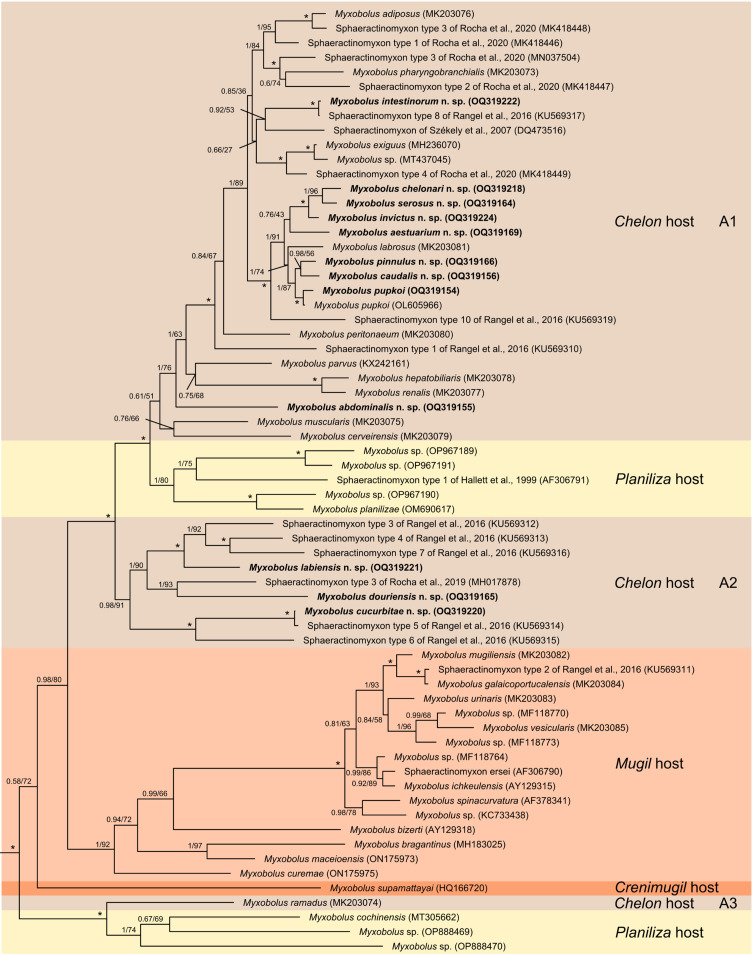
Bayesian inference topology displaying the position of the novel 18S rDNA sequences (in bold) within the clade of mugiliform-infecting myxobolids. Node values are Bayesian inference probabilities and maximum likelihood bootstrap values; asterisks indicate full support in both methodologies.

Bayesian inference and maximum likelihood retrieved highly congruent tree topologies. Both analyses revealed the 18S rDNA sequences of the novel *Myxobolus* spp. clustering within *Chelon*-infecting lineages (Fig. 5). *Myxobolus pinnula* n. sp., *M. caudalis* n. sp., *M. chelonari* n. sp., *M. aestuarium* n. sp., *M. invictus* n. sp., *M. peritonaei* n. sp., *M. abdominalis* n. sp., *M. intestinicola* n. sp., and the novel sequence of *M. pupkoi* clustered within the subclades of the main *Chelon*-infecting lineage (Fig. 5, clade A1). The novel sequence of *M. pupkoi* specifically clustered with its conspecific sequence from the original species description (OL605966), while *M. intestinicola* n. sp. clustered together with its actinospore counterpart, the Sphaeractinomyxon type 8 of Rangel *et al*. [38] (KU569317). *Myxobolus cucurbitiformis* n. sp., *M. douroensis* n. sp., and *M. labicola* n. sp. clustered together with several sphaeractinomyxon types to form a novel independent, smaller *Chelon*-infecting lineage (Fig. 5, clade A2). *Myxobolus cucurbitiformis* n. sp. specifically clustered with the Sphaeractinomyxon type 5 of Rangel *et al.* [38] (KU569314), here determined as its actinospore counterpart. In accordance with previous cladograms [21, 40], *M. ramadus* from the gills of *C. ramada* was retrieved at a more basal position (Fig. 5, clade A3), and is here shown as a sister branch to a small lineage of *Myxobolus* spp. that infect hosts of the genus *Planiliza*. A second lineage of *Planiliza*-infecting *Myxobolus* consistently branched at the basis of the most derived lineage of *Chelon*-infecting species. Finally, all *Myxobolus* spp. that infect hosts of the genus *Mugil* clustered together within the same lineage, while *M. supamattayai* formed a single branch as the sole representative of *Crenimugil*-infecting species (Fig. 5).

## Discussion

Presently, it is widely accepted that the combination of several criteria, such as myxospore morphology and morphometry, host and tissue specificity/tropism, geographical location/habitat, and molecular data, is required for the reliable description of myxozoan species [2, 19]. Recent studies showed that, in the case of mugiliform-infecting *Myxobolus*, molecular data are mandatory for species identification, due to the notable lack of distinctive morphological characters that could allow myxospore differentiation [39, 40]. Congruently, the *Myxobolus* spp. described in this study lacked significant morphologically distinguishable features allowing reliable differentiation amongst each other and in relation to other closely related species, thus reinforcing the need to perform sequencing of selected genetic markers whenever working with mugiliform-infecting *Myxobolus.* Morphology-based comparisons, however, remained necessary to establish differentiation from known mugiliform-infecting *Myxobolus* species that lack molecular data.

Worldwide, the thicklip grey mullet *C. labrosus* has been reported to host 5 *Myxobolus* spp. [39, 40], including *M. peritoneum* and *M. labrosus* described from the peritoneum and urinary bladder of specimens caught from the Minho River estuary, in northern Portugal [40]. In this study, 11 new *Myxobolus* spp. are described from specimens obtained from the Douro River estuary, also located in northern Portugal. This species richness suggests that the real diversity of *Myxobolus* infecting *C. labrosus* is probably extremely underestimated. Although the *Myxobolus* community infecting this host varied between geographically close estuaries (the Douro and Minho River estuaries are less than 85 km apart), it should be considered that the study performed here was not ecologically significant given that seasonality was not evaluated. Therefore, a more thorough and organized effort in sampling *C. labrosus* specimens will most likely reveal even higher biodiversity of *Myxobolus*, with some species probably present in more than a single estuary. This hypothesis is supported by the matching of *M. cucurbitiformis* n. sp. and *M. intestinicola* n. sp. with Sphaeractinomyxon type 5 and Sphaeractinomyxon type 8, respectively reported by Rangel *et al.* [38] from the Aveiro estuary, 84 km south of the Douro estuary. In this study, the occurrence of highest species diversity and overall prevalence of infection in the gills agrees with previous studies that report this organ as a preferential site of infection for myxobolids, potentially allowing optimal myxospore release [14, 31, 32].

Even more patchy than our knowledge of the biodiversity of *Myxobolus* parasites, is that of their life cycles. The information presently available links this genus to a significant diversity of actinospore morphotypes [13]; however, a specific correspondence of mugiliform-infecting species to the sphaeractinomyxon collective group was recently proposed by Rocha *et al.* [42, 43]. In this study, the molecular matching of *M. cucurbitiformis* n. sp. and *M. intestinicola* n. sp. with Sphaeractinomyxon type 5 and Sphaeractinomyxon type 8 of Rangel *et al.* [38], respectively reinforces the conjecture that sphaeractinomyxon types constitute specific life cycle counterparts of mugiliform-infecting *Myxobolus*.

A 99.3% similarity score matched the sequence of one *Myxobolus* isolate from the gills of *C. labrosus* with that of *M. pupkoi* (OL605966) reported from the gill arches of *C. ramada* in the Galilee Sea off Israel [21], despite morphological comparisons showing significant differences between these geographical isolates (see Table 3). Although genetic similarity higher than 98% is usually considered to be representative of intraspecific variation among myxozoans [17], the boundary between myxobolid intraspecific and interspecific variability is not clear; there is no exact value of genetic similarity in the 18S rDNA sequences that determines whether two isolates can be discriminated as being conspecific or congener. Numerous instances of high values of intraspecific variation have been reported in myxobolids, namely *M. koi* (3.0%), *H. corruscans* (2.3%), *H. maculosus* (1.9%), *M. flavus* (1.9%), *M. dujardini* (3.6%), and *M. ellipsoides* (1.9%) [4, 6, 34]. Likewise, low values of interspecific variability have also been reported for *M. pseudodispar*, *M. musculi*, and *M. cyprini*, which ranged between 0.3 and 0.6% of genetic difference; *M. pendula* and *M. pellicides* with 0.4%; *M. fryeri* and *M. insidiosus* with 0.5%; among others [7, 11, 16, 24, 33]. Since characters like morphology, host specificity, tissue tropism, and geographical location all come into play when inferring about intra- or interspecific variation, the decision of determining genetic differences as being representative of one or the other must be made individually. In the case of *M. pupkoi*, the small genetic difference determined between geographic isolates (0.7%) mostly took place at the 3′ end of the 18S rDNA sequence and, therefore, cannot confidently be taken as a premise of interspecific variability. Furthermore, our isolate shares the same site of infection and host genus with the Israeli isolate, with Gupta *et al.* [21] even proposing that *M. pupkoi* was introduced into the Sea of Galilee via the Mediterranean Sea. Thus, the only considerable difference between our isolate and the Israeli isolate is myxospore morphometry, given that the myxospores observed in this study significantly surpassed the size range reported by Gupta *et al.* [21]. However, morphological plasticity is not uncommon between myxozoans, and has been reported between geographical isolates of several *Myxobolus* spp. [20, 21, 26, 47, 49, 51]. For instance, in clear parallelism to our case, Israeli isolates of *Myxobolus exiguus* Thélohan, 1895 were also reported to have smaller myxospores than Portuguese isolates belonging to the same species, with identification based on the comparison of ~2,000 bp sequences [21]. Thus, the genetic difference determined here between geographical isolates of *M. pupkoi* is proposed to be representative of intraspecific variability, with this species displaying morphological plasticity that may be hypothesized to correlate with adaptation to distinct environmental pressures and invertebrate communities.

In agreement with previous cladograms (e.g. [8, 21, 40]), our phylogenetic analysis showed all novel *Myxobolus* sequences clustering within the clade of mugiliform-infecting *Myxobolus*, which reinforces the proposed monophyletic origin of this group [40]. Congruently with host affinity being the main evolutionary driver of myxobolid radiation [6, 27], host-associated clustering was retrieved within the mugiliform-infecting clade, which included well-defined lineages of species parasitizing mullets belonging to the genera *Chelon*, *Mugil*, *Planiliza*, and *Crenimugil*. The identification of more than one *Chelon*-infecting lineage suggests that myxobolids acquired this fish genus as secondary hosts multiple times during their evolution. The same can be hypothesized for *Planiliza*-infecting species, which appear to be included within two separate lineages. Finally, the considerably high number of unmatched sphaeractinomyxon types included in the most derived *Chelon*-infecting lineages strengthens our contention that *Myxobolus* diversity in *C. labrosus* most likely remains underestimated. This shows a clear need to perform further studies targeting not only this species, but also others of the genus *Chelon*, and mullets in general.

## References

[1] Almeida PR. 2003. Feeding ecology of *Liza ramada* (Risso, 1810) (Pisces, Mugilidae) in a south-western estuary of Portugal. Estuarine, Coastal and Shelf Science, 57, 313–323.

[2] Atkinson SD, Bartošová-Sojková P, Whipps CM, Bartholomew JL. 2015. Approaches for characterising myxozoan species, in Myxozoan evolution, ecology and development. Okamura B, Gruhl A, Bartholomew JL, Editors. Springer International Publishing: Switzerland. p. 111–123.

[3] Bartošová P, Fiala I, Hypša V. 2009. Concatenated SSU and LSU rDNA data confirm the main evolutionary trends within myxosporeans (Myxozoa: Myxosporea) and provide and effective tool for their molecular phylogenetics. Molecular Phylogenetics and Evolution, 53, 81–93. 19477283 10.1016/j.ympev.2009.05.018

[4] Camus AC, Griffin MJ. 2010. Molecular characterization and histopathology of *Myxobolus koi* infecting the gills of a koi, *Cyprinus carpio*, with an amended morphological description of the agent. Journal of Parasitology, 96, 116–124. 19821634 10.1645/GE-2113.1

[5] Cardona L. 2001. Non-competitive coexistence between Mediterranean grey mullet: Evidence from seasonal changes in food availability, niche breadth and trophic overlap. Journal of Fish Biology, 59, 729–744.

[6] Carriero MM, Adriano EA, Silva MR, Ceccarelli PS, Maia A. 2013. Molecular phylogeny of the *Myxobolus* and *Henneguya* genera with several new South American species. PLoS One, 8, e73713. 24040037 10.1371/journal.pone.0073713 PMC3764033

[7] Cech G, Borzák R, Molnár K, Székely C. 2015. Three new species of *Myxobolus* Bütschli, 1882 (Myxozoa: Myxobolidae) infecting the common nase *Chondrostoma nasus* (L.) in the River Danube. Systematic Parasitology, 92, 101–111. 26358070 10.1007/s11230-015-9589-5

[8] Correya MS, Pananghat V, Karayi SN. 2022. Morphological and molecular characterization of *Myxobolus planilizae* n. sp. (Cnidaria; Myxosporea; Myxobolidae) infecting the largescale mullet *Planiliza macrolepis* (Smith, 1846) collected from Cochin backwaters, India. Acta Parasitologica, 68, 42–50. 36348180 10.1007/s11686-022-00637-y

[9] Das M. 1996. Myxozoan and urceolarid ciliate parasites of wild and cultured *Liza parsia* in deltaic West Bengal. Journal of the Inland Fisheries Society of India, 28, 46–56.

[10] Dorothy KP, Kalavati C. 1992. Two new myxosporean parasites of the mullet *Liza macrolepis* (Smith). Uttar Pradesh Journal of Zoology, 12, 15–19.

[11] Easy RH, Johnson SC, Cone DK. 2005. Morphological and molecular comparison of *Myxobolus procerus* (Kudo, 1934) and *M. intramusculi* n. sp. (Myxozoa) parasitizing muscles of the trout-perch *Percopsis omiscomaycus*. Systematic Parasitology, 61, 115–122. 15980965 10.1007/s11230-005-3135-9

[12] Eiras JC, Molnár K, Lu Y. 2005. Synopsis of the species of *Myxobolus* Bütschli, 1882 (Myxozoa: Myxosporea: Myxobolidae). Systematic Parasitology, 61, 1–46. 15928990 10.1007/s11230-004-6343-9

[13] Eszterbauer E, Atkinson S, Diamant A, Morris D, El-Matbouli M, Hartikainen H. 2015. Myxozoan life cycles: practical approaches and insights, in Myxozoan Evolution, Ecology and Development. Okamura B, Gruhl A, Bartholomew JL, Editors. Springer International Publishing: Switzerland. p. 175–198.

[14] Eszterbauer E, Sipos D, Forró B, Holzer AS. 2013. Molecular characterization of *Sphaerospora molnari* (Myxozoa), the agent of gill sphaerosporosis in common carp *Cyprinus carpio carpio*. Diseases of Aquatic Organisms, 104, 59–67. 23670080 10.3354/dao02584

[15] Faye N, Kpatcha TK, Debakate C, Fall M, Toguebaye BS. 1999. Gill infections due to myxosporean (Myxozoa) parasites in fishes from Senegal with description of *Myxobolus hani* sp. n. Bulletin of the European Association of Fish Pathologists, 19, 14–16.

[16] Ferguson JA, Atkinson SD, Whipps CM, Kent ML. 2008. Molecular and morphological analysis of *Myxobolus* spp. of salmonid fishes with the description of a new *Myxobolus* species. Journal of Parasitology, 94, 1322–1334. 19127969 10.1645/GE-1606.1

[17] Fiala I. 2006. The phylogeny of Myxosporea (Myxozoa) based on small subunit ribosomal RNA gene analysis. International Journal of Parasitology, 36, 1521–1534. 16904677 10.1016/j.ijpara.2006.06.016

[18] Fiala I, Bartošová P. 2010. History of myxozoan character evolution on the basis of rDNA and EF-2 data. BMC Evolutionary Biology, 10, 1–13. 20667097 10.1186/1471-2148-10-228 PMC2927925

[19] Fiala I, Bartošová-Sojková P, Whipps CM. 2015. Classification and phylogenetics of Myxozoa, in Myxozoan evolution, ecology and development. Okamura B, Gruhl A, Bartholomew JL, Editors. Springer International Publishing: Switzerland. p. 85–110.

[20] Guo Q, Huang M, Liu Y, Zhang X, Gu Z. 2018. Morphological plasticity in *Myxobolus* Bütschli, 1882: a taxonomic dilemma case and renaming of a parasite species of the common carp. Parasites & Vectors, 11, 399. 29986743 10.1186/s13071-018-2943-0 PMC6038286

[21] Gupta A, Haddas-Sasson M, Gayer K, Huchon D. 2022. Myxozoan infection in thinlip mullet *Chelon ramada* (Mugiliformes: Mugilidae) in the Sea of Galilee. Scientific Reports, 12, 10049. 35710685 10.1038/s41598-022-13215-z PMC9203526

[22] Hallett SL, Diamant A. 2001. Ultrastructure and small-subunit ribosomal DNA sequence of *Henneguya lesteri* n. sp. (Myxosporea), a parasite of sand whiting *Sillago analis* (Sillaginidae) from the coast of Queensland. Australia. Diseases of Aquatic Organisms, 46, 197–212. 11710554 10.3354/dao046197

[23] Hillis DM, Dixon MT. 1991. Ribosomal DNA: molecular evolution and phylogenetic inference. Quarterly Review of Biology, 66, 411–453. 1784710 10.1086/417338

[24] Kent ML, Andree KB, Bartholomew JL, El-Matbouli M, Desser SS, Devlin RH, Feist SW, Hedrick RP, Hoffmann RW, Khattra J, Hallett SL, Lester RJ, Longshaw M, Palenzeula O, Siddall ME, Xiao C. 2001. Recent advances in our knowledge of the Myxozoa. Journal of Eukaryotic Microbiology, 48, 395–413. 11456316 10.1111/j.1550-7408.2001.tb00173.x

[25] Laffaille P, Feunteun E, Lefebvre C, Radureau A, Sagan G, Lefeuvre JC. 2002. Can thin-lipped mullet directly exploit the primary and detritic production of European macrotidal salt marshes? Estuarine, Coastal and Shelf Science, 54, 729–736.

[26] Liu XH, Batueva MD, Zhao YL, Zhang JY, Zhang QQ, Li TT, Li AH. 2016. Morphological and molecular characterisation of *Myxobolus pronini* n. sp. (Myxozoa: Myxobolidae) from the abdominal cavity and visceral serous membranes of the gibel carp *Carassius auratus gibelio* (Bloch) in Russia and China. Parasites & Vectors, 9, 562. 27782848 10.1186/s13071-016-1836-3 PMC5080772

[27] Liu Y, Lövy A, Gu Z, Fiala I. 2019. Phylogeny of Myxobolidae (Myxozoa) and the evolution of myxospore appendages in the *Myxobolus* clade. International Journal of Parasitology, 49, 523–530. 31077679 10.1016/j.ijpara.2019.02.009

[28] Liu Y, Whipps CM, Gu ZM, Zeng LB. 2010. *Myxobolus turpisrotundus* (Myxosporea: Bivalvulida) spores with caudal appendages: investigating the validity of the genus *Henneguya* with morphological and molecular evidence. Parasitology Research, 107, 699–706. 20512504 10.1007/s00436-010-1924-9

[29] Lom J, Arthur JR. 1989. A guideline for the preparation of species descriptions in Myxosporea. Journal of Fish Diseases, 12, 151–156.

[30] Lom J, Dyková I. 2006. Myxozoan genera: definition and notes on taxonomy, life-cycle terminology and pathogenic species. Folia Parasitologica, 53, 1–36. 16696428

[31] Molnár K. 2002. Site preference of fish myxosporeans in the gill. Diseases of Aquatic Organisms, 48, 197–207. 12033706 10.3354/dao048197

[32] Molnár K, Eszterbauer E. 2015. Specificity of infection sites in vertebrate hosts, in Myxozoan evolution, ecology and development. Okamura B, Gruhl A, Bartholomew JL, Editors. Springer International Publishing: Switzerland. p. 295–313.

[33] Molnár K, Eszterbauer E, Székely C, Dán Á, Harrach B. 2002. Morphological and molecular biological studies on intramuscular *Myxobolus* spp. of cyprinid fish. Journal of Fish Diseases, 25, 643–652.

[34] Molnár K, Marton S, Eszterbauer E, Székely C. 2006. Comparative morphological and molecular studies on *Myxobolus* spp. infecting chub from the River Danube, Hungary, and description of *M. muellericus* sp. n. Diseases of Aquatic Organisms, 73, 49–61. 17240752 10.3354/dao073049

[35] Nei M, Kumar S. 2000. Molecular evolution and phylogenetics. Oxford University Press: Oxford, USA.

[36] Okamura B, Gruhl A, Bartholomew JL. 2015. An introduction to myxozoan evolution, ecology and development, in Myxozoan evolution, ecology and development. Okamura B, Gruhl A, Bartholomew JL, Editors. Springer International Publishing: Switzerland. p. 1–20.

[37] Ovcharenko M. 2015. Microparasites of worldwide mullets. Annals of Parasitology, 61, 229–239. 26878619 10.17420/ap6104.12

[38] Rangel LF, Castro R, Rocha S, Cech G, Casal G, Azevedo C, Székely C, Cavaleiro F, Santos MJ. 2016. Description of new types of sphaeractinomyxon actinospores (Myxozoa: Myxosporea) from marine tubificid oligochaetes, with a discussion on the validity of the tetraspora and the endocapsa as actinospore collective group names. Parasitology Research, 115, 2341–2351. 26969178 10.1007/s00436-016-4983-8

[39] Rocha S, Azevedo C, Oliveira E, Alves Â, Antunes C, Rodrigues P, Casal G. 2019. Phylogeny and comprehensive revision of mugiliform-infecting myxobolids (Myxozoa, Myxobolidae), with the morphological and molecular redescription of the cryptic species *Myxobolus exiguus*. Parasitology, 146, 479–496. 30301476 10.1017/S0031182018001671

[40] Rocha S, Casal G, Alves Â, Antunes C, Rodrigues P, Azevedo C. 2019. Myxozoan biodiversity in mullets (Teleostei, Mugilidae) unravels hyperdiversification of *Myxobolus* (Cnidaria, Myxosporea). Parasitology Research, 118, 3279–3305. 31673834 10.1007/s00436-019-06476-7

[41] Rocha S, Casal G, Garcia P, Matos E, Al-Quraishy S, Azevedo C. 2014. Ultrastructure and phylogeny of the parasite *Henneguya carolina* sp. nov. (Myxozoa), from the marine fish *Trachinotus carolinus* in Brazil. Diseases of Aquatic Organisms, 112, 139–148. 25449325 10.3354/dao02794

[42] Rocha S, Rangel LF, Casal G, Azevedo C, Rodrigues P, Santos MJ. 2020. Involvement of sphaeractinomyxon in the life cycle of mugiliform-infecting *Myxobolus* (Cnidaria, Myxosporea) reveals high functionality of actinospore morphotype in promoting transmission. Parasitology, 147, 1320–1329. 32594944 10.1017/S0031182020001043 PMC10317763

[43] Rocha S, Rangel LF, Castro R, Severino R, Azevedo C, Santos MJ, Casal G. 2019. The potential role of the sphaeractinomyxon collective group (Cnidaria, Myxozoa) in the life cycle of mugiliform-infecting myxobolids, with the morphological and molecular description of three new types from the oligochaete *Tubificoides insularis*. Journal of Invertebrate Pathology, 160, 33–42. 30521780 10.1016/j.jip.2018.12.001

[44] Ronquist F, Huelsenbeck JP. 2003. MrBayes 3: Bayesian phylogenetic inference under mixed models. Bioinformatics, 19, 1572–1574. 12912839 10.1093/bioinformatics/btg180

[45] Sharon G, Ucko M, Tamir B, Diamant A. 2019. Co-existence of *Myxobolus* spp. (Myxozoa) in gray mullet (*Mugil cephalus*) juveniles from the Mediterranean Sea. Parasitology Research, 118, 159–167. 30499010 10.1007/s00436-018-6151-9

[46] Shen K, Durand JD. 2016. The biogeography of Mugilidae in India, South-East and East Asia, in Biology, ecology and culture of grey mullets (Mugilidae). Crosetti D, Blaber S, Editors. CRC Press: Florida, USA. p. 63–84.

[47] Tahir UB, Guo Q, Gu Z. 2021. Fins infestation induced by *Myxobolus xiantaoensis* in yellow catfish *Tachysurus fulvidraco* Richardson, 1846: some pathophysiological and molecular insights. Microbial Pathogenesis, 153, 104772. 33529735 10.1016/j.micpath.2021.104772

[48] Tamura K, Stecher G, Kumar S. 2021. MEGA 11: Molecular evolutionary genetics analysis version 11. Molecular Biology and Evolution, 38, 3022–3027. 33892491 10.1093/molbev/msab120 PMC8233496

[49] Urawa S, Freeman MA, Johnson SC, Jones SR, Yokoyama H. 2011. Geographical variation in spore morphology, gene sequences, and host specificity of *Myxobolus arcticus* (Myxozoa) infecting salmonid nerve tissues. Diseases of Aquatic Organisms, 96, 229–237. 22132501 10.3354/dao02398

[50] Whipps CM, Adlard RD, Bryant MS, Lester RJ, Findlay V, Kent ML. 2003. First report of three *Kudoa* species from eastern Australia: *Kudoa thyrsites* from mahi mahi (*Coryphaena hippurus*), *Kudoa amamiensis* and *Kudoa minithyrsites* n. sp. from sweeper (*Pempheris ypsilychnus*). Journal of Eukaryotic Microbiology, 50, 215–219. 12836879 10.1111/j.1550-7408.2003.tb00120.x

[51] Yokoyama H, Suzuki J, Shirakashi S. 2014. *Kudoa hexapunctata* n. sp. (Myxozoa: Multivalvulida) from the somatic muscle of Pacific bluefin tuna *Thunnus orientalis* and re-description of *K. neothunni* in yellowfin tuna *T. albacares*. Parasitology International, 63, 571–579. 24709084 10.1016/j.parint.2014.03.006

[52] Yurakhno VM, Ovcharenko MO. 2014. Study of Myxosporea (Myxozoa) infecting worldwide mullets with description of a new species. Parasitology Research, 113, 3661–3674. 25070577 10.1007/s00436-014-4031-5 PMC4172997

